# Identification of lung cancer gene markers through kernel maximum mean discrepancy and information entropy

**DOI:** 10.1186/s12920-019-0630-4

**Published:** 2019-12-20

**Authors:** Zhixun Zhao, Hui Peng, Xiaocai Zhang, Yi Zheng, Fang Chen, Liang Fang, Jinyan Li

**Affiliations:** 1 Advanced Analytics Institute, Faculty of Engineering and Information Technology, University of Technology Sydney, PO Box 123, Broadway, Sydney, 2007 NSW Australia; 2 Faculty of Engineering and Information Technology, University of Technology Sydney, PO Box 123, Broadway, Sydney, 2007 NSW Australia; 3 School of Computer, National University of Defense Technology, Changsha, 410073 China

**Keywords:** Lung cancer, Maximum mean discrepancy, Information theory, Biomarker discovery

## Abstract

**Background:**

The early diagnosis of lung cancer has been a critical problem in clinical practice for a long time and identifying differentially expressed gene as disease marker is a promising solution. However, the most existing gene differential expression analysis (DEA) methods have two main drawbacks: First, these methods are based on fixed statistical hypotheses and not always effective; Second, these methods can not identify a certain expression level boundary when there is no obvious expression level gap between control and experiment groups.

**Methods:**

This paper proposed a novel approach to identify marker genes and gene expression level boundary for lung cancer. By calculating a kernel maximum mean discrepancy, our method can evaluate the expression differences between normal, normal adjacent to tumor (NAT) and tumor samples. For the potential marker genes, the expression level boundaries among different groups are defined with the information entropy method.

**Results:**

Compared with two conventional methods t-test and fold change, the top average ranked genes selected by our method can achieve better performance under all metrics in the 10-fold cross-validation. Then GO and KEGG enrichment analysis are conducted to explore the biological function of the top 100 ranked genes. At last, we choose the top 10 average ranked genes as lung cancer markers and their expression boundaries are calculated and reported.

**Conclusion:**

The proposed approach is effective to identify gene markers for lung cancer diagnosis. It is not only more accurate than conventional DEA methods but also provides a reliable method to identify the gene expression level boundaries.

## Background

Small-cell lung carcinoma (SCLC) and non-small-cell lung carcinoma (NSCLC) are two main types of lung cancer, comprising the majority of clinic cases [1]. As the most common cancer, lung cancer is the leading cause of cancer-related deaths all over the world [2, 3]. However, most lung cancer cases were diagnosed in a very late stage when symptoms like coughing, coughing up blood, shortness of breath and chest pains appeared. Many early-diagnosed lung cancer cases were detected by accident [3, 4]. In the clinic practice, the most widely used examinations for lung cancer are chest radiography and computed tomography(CT), but these two methods require visible and irreversible histological variants in human lung, resulting in rather low sensitivity in the early stage [5–7]. Therefore, it is a crucial issue to find more timely and accurate approaches for lung cancer early-stage diagnosis.

Due to the progress in molecular biology, some molecules which play vital roles in lung cancer development are possible to diagnose cancer and distinguish the specific cancer sub-types [8–10]. Researchers have explored to identify efficient biomarkers from these molecules as the indicator of the pathogenic process to improve the diagnosis sensitivity [11]. These explorations are mainly focused on genetic mutations, DNA methylation profile, miRNA synthesis profile and especially blood proteins [12–19]. Till now, panels of protein markers have been identified and intensively used in clinic applications. For example, the combinations of CEACAM, CYFRA 21-1, ProGRP, CA125, NSE (neuron-specific enolase) and NY-ESO (cancer-testis antigen) are popular lung cancer diagnosis markers [20–24]. Recently, researchers also discovered that *β*-chain of human haptoglobin [25], SAA (serum amyloid A) [26], APOA1 (apolipoprotein A-1) [27] and some other proteins [28] may be potential biomarkers. Despite the advances in protein marker discovery, some disadvantages of protein markers are still existing, like genetic heterogeneity of tumors, poor reproducibility of laboratory test and low concentration of the proteins [18, 29]. Recent years the next-generation sequence technologies have promoted the study of disease-related genomes. Projects like The Cancer Genome Atlas (TCGA) [30] and the Genotype-Tissue Expression (GTEx) [31] have collected a large number of sequencing experiments and provided tissue-specific gene expression data in public. As some genes have distinct expression levels between normal and tumor tissues for the reason of disease development, they are promising to diagnose lung cancer more timely and accurately.

During the past years, gene differential expression analysis (DEA) has been extensively applied in the preprocess of high-throughput profiling data collected from microarrays [32–34]. Based on statistical models, researchers developed tools to identify genes which had distinct expression levels between different experiment groups. Compared with the microarray data, the RNA-seq raw data comes with the unique feature of discrete reads which should be analyzed under an appropriate statistical hypothesis [35]. According to the statistical hypothesis, the existing RNA-seq analysis models can be categorized into Poisson model, negative binomial model, beta-binomial model, and Bayesian model [36, 37]. These models can tell whether the gene expression levels are the same between experiment groups and calculate a confidence coefficient scores (also named *p*-value) suggesting the magnitude of expression difference.

In cancer studies, the histologically normal tissue adjacent to tumor is usually used to compare with the tumor tissue under the assumption that they are the same with real healthy tissues This approach allows researchers to compare samples from the same patient and reduce the individual specific effects. However, recent studies have deepened our understanding about NAT tissue, indicating that NAT is not exactly equal to the real healthy tissue [38]. In NAT tissues, the specific micro-environment surrounding tumor makes the change of gene expression in various pathways that are related to disease development. In order to identify efficient and meaningful marker genes, we proposed to detect differentially expressed genes(DEGs) from real normal, NAT and tumor tissues.

Here, we present a novel approach to identify genes markers for lung cancer with kernel maximum mean discrepancy (MMD) and Information Entropy. As mentioned above, the conventional DEA methods can calculate a *p*-value to evaluate the expression difference based on certain statistical hypothesis, but it’s hard to decide which distribution assumption is correct before calculation. Inspired by the distribution measure method of transfer learning, we use the kernal MMD to detect DEGs between tumor, NAT and normal tissues. This method can output the maximum mean discrepancy score which indicates the degree of differential expression which does not require a statistical hypothesis on data distribution. Besides, although the *p*-value of conventional techniques can identify DEGs, it is essential to define a threshold of expression level to distinguish different types of tissue. Commonly, Researchers would like to take the upper boundary of lower expressed tissue or lower edge of higher expressed tissue as the threshold when there is a distinct expression gap. But this kind of gap is not always existing and then the threshold is hard to define. As the gene expression level is continuous data and how to choose a definite threshold point is a tough task. Here we applied the information theory to solve this problem.

In this paper, we firstly evaluate the expression level difference of 23368 genes in normal, normal adjacent tumor and tumor tissues with the kernel maximum mean discrepancy. Then the top-ranked genes selected by kernel MMD method are compared with genes selected by two conventional DEA methods, t-test and fold change. Then GO and KEGG pathway enrichment analysis are conducted to analyze the top 100 genes ranked by average MMD scores. Lastly, the top 10 genes are selected as marker genes for lung cancer and their expression boundaries between normal, NAT and tumor tissues are identified by the proposed information theory method.

## Methods

### Dataset

Three gene expression datasets used in this paper are collected from different tissue types in reference [38], containing the expression data of 23368 genes. Dataset 1 includes the gene expression data of 373 normal healthy samples. The raw reads file of dataset 1 is obtained from the GTEx program (phs000424.v6.p1, 18 November 2015). Dataset 2 has 59 NAT tissues, while dataset 3 has 541 lung cancer tumor tissues. Their raw feature counts and FPKM values are original from NCBI Gene Expression Omnibus (GEO) [39]. Since the raw values are from different data sources, the RNA-sequencing raw reads files were processed and normalized with the Rsubread package and aligned to the UCSC hg19 reference genome with the same pipeline. The processed GTEx expression profiles of dataset 1 are available in GEO under an accession number GSE86354 and other two datasets are deposited as GSE62944.

### Gene marker identification framework

With the above three datasets, we apply a novel approach to detect DEGs and determine the expression boundaries between normal, NAT and tumor cells as the criterion of lung cancer diagnosis.

**Fig. 1:**
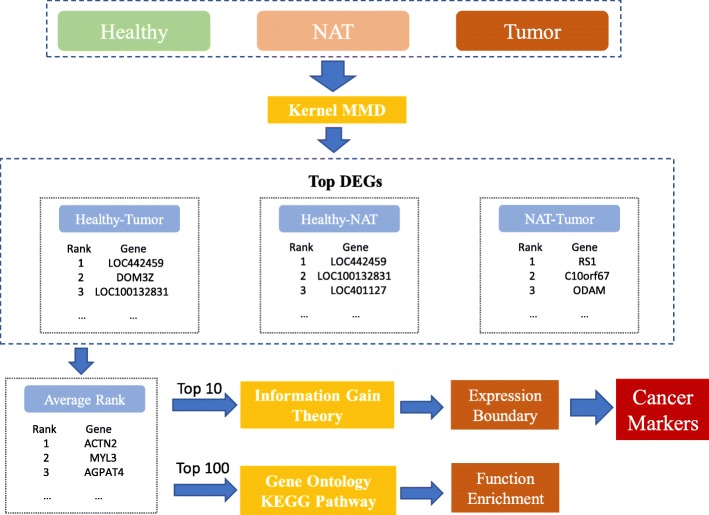
Gene marker identification framework

In our method, there are mainly four steps: First, the kernel Maximum Mean Discrepancy is used to identify DEGs between two types of tissues respectively and genes are ranked by the MMD values; Second, the genes with top average MMD rankings are selected from all genes; Third, genes selected from the previous step are put into KEGG pathway analysis and GO enrichment analysis to validate the efficiency of those gene markers; Last, we define the gene expression boundaries for the top 10 marker genes with information gain theory. The whole framework of the proposed approach is illustrated in Fig. 1.

### Kernel maximum mean discrepancy

The problem of comparing the probability distribution between two sample groups, also referred to as two-sample problem, widely exists in data science areas. In bioinformatics field, this problem is extensively existing in micro-array data analysis, database attribute matching, data integration from different platforms and so on. The key point of two-sample problem is how to determine if two groups of observations are from the same distribution and some statistical test methods were applied to address that in previous researches.

However, these methods have different statistical modelings based on specific assumptions of data distribution, which is commonly unknown before calculation in practical use. In some previous studies, researchers have explored to using the kernel Maximum Mean Discrepancy (MMD) method to test the distribution difference in RNA-Transcript expression and pathway differential expression and achieved better performance than traditional statistical tests [40, 41].Here, we adopt kernal MMD to identify the DEGs in lung cancer gene expression data.

Give *F* to be a class of functions *f*:*χ*→*R*. Two samples *X*={*x*_1_,*x*_2_,…*x*_*m*_} and *Y*={*y*_1_,*y*_2_,…*y*_*n*_} are drawn form two probability distribution *p* and *q*, respectively. The empirical estimation of MMD value is as following [42]:

1 $$ MMD[F,p,q]:= \sup_{f\in F}\left(E_{p}[f(x)]-E_{q}[f(y)]\right)  $$

2 $$ {}MMD[F,p,q]:= \sup_{f\in F}\left(\frac{1}{m}\sum_{i=1}^{m}F(x_{i})-\frac{1}{n}\sum_{i=1}^{n}F(y_{i})\right)  $$

As the definition above, if the function class F is rich enough, the value of MMD will be zero if and only if *p=q*. But a too rich F will lead to that MMD differs from zero for most finite sample estimates. Thus some restrictions ought to be placed on the function class. One trade-off way is to set F as the unit ball in a universal reproducing kernel Hilbert space *H*, defined on the compact metric space *χ*. Since H is a complete inner product space of functions *f*:*χ*→*R*, the function mapping *f*→*f*(*x*) can be expressed as an inner product via *f*(*x*)=〈*f*,*ϕ*(*x*)〉_*H*_,where *ϕ*:*χ*→*H* is the feature space map from *x* to *H*. Then MMD can be rewritten as:

3 $$ {{}\begin{aligned} MMD[F,p,q]&= \sup_{\left \| f \right \|_{H}\leq 1}E_{p}[f(x)]-E_{q}[f(y)]\\ &=\sup_{\left \| f \right \|_{H}\leq 1}E_{p}\left[\left \langle f,\phi (x) \right \rangle_{H}\right]-E_{q} \left[\left \langle f,\phi (y) \right \rangle_{H} \right]\\ &=\sup_{\left \| f \right \|_{H}\leq 1}\left \langle \mu_{p}- \mu_{q}, f\right \rangle_{H}\\ &=\left \| \mu_{p}- \mu_{q} \right \|_{H} \end{aligned}}  $$

Then we can calculate like the following function:

4 $$ \begin{aligned} MMD^{2}&=\left \langle \mu_{p}- \mu_{q},\mu_{p}- \mu_{q} \right \rangle_{H}\\ &=\left \langle \mu_{p},\mu_{p} \right \rangle_{H}+\left \langle \mu_{q},\mu_{q} \right \rangle_{H}-2\left \langle \mu_{p},\mu_{q} \right \rangle_{H}\\ &=E_{p}\left \langle \phi (x),\phi (x^{\prime}) \right \rangle_{H}+E_{p}\left \langle \phi (y),\phi (y^{\prime}) \right \rangle_{H}\\ &\quad-2E_{p,q}\left \langle \phi (x),\phi (y) \right \rangle_{H} \end{aligned}  $$

As the inner product can be replaced by Gaussian kernel *k*(*x,x*^′^)=*exp*(−∥*x*−*x*^′^∥^2^/(2*σ*^2^)),the value of *MMD*^2^ can be figured out as:

5 $$ \begin{aligned} MMD^{2}&=\frac{1}{m(m-1)}\sum_{i\neq j}^{m}k(x_{i},x_{j})-\frac{2}{mn}\sum_{i,j=1}^{m,n}k(x_{i},y_{j})\\ &+\frac{1}{n(n-1)}\sum_{i\neq j}^{m}k(y_{i},y_{j}) \end{aligned}  $$

In our method, the minimum variance unbiased estimate of MMD value is obtain according to the above functions based on Shogun package in python [43]. The computational complexity of MMD method is *O*(*n*^2^). The MMD score can evaluate the gene expression difference between different sample types, while a higher MMD score means greater gene expression level difference.

### Boundary discovery method

As a biomarker, there should be an expression threshold for the marker gene as the indicator for disease diagnosis. If the gene expression level is proved to be different in normal and tumor tissues, it is necessary to define a threshold of expression level as the boundary. When the gene expression level has a distinct gap between normal and tumor samples, the threshold is commonly the lower or upper boundary of this gap. However, the expression level does not have that kind of obvious gap all the time, thus how to define a reliable boundary is challenging in these cases.

Here we propose to identify the threshold with information theory which has been widely used in decision tree algorithms for classification problems. According to the information theory, the change of information entropy which is also named information gain can evaluate the classification efficiency of a threshold point. If there is the expression data of a gene from m normal samples and n tumor samples in dataset D, *p*_*m*_ and *p*_*n*_ refer to the proportions of normal and tumor samples in all samples, then the original entropy of D is defined as:

6 $$ Ent(D)=-\sum_{k=m,n}^{} p_{k} \log_{2} p_{k}  $$

In the boundary identification, all samples are re-classified by the gene expression level with a split point of x and *D*^*v*^ denotes the new dataset re-classed by x. Then the information gain of this split point can be computed as:

7 $$ {Gain}(D, x)=Ent(D)-\sum_{v=1}^{2} \frac{\left|D^{v}\right|}{|D|}{Ent}\left(D^{v}\right)  $$

Different from discrete data, the expression level is continuous and it is inappropriate to use the expression level values in samples as the split points. Besides, as the distribution of the expression level is also unknown, we cannot use the probability function to calculate the entropy. To address this problem, we propose to deal with continuous data like discrete data: First, the expression level values are sorted from small to large and the middle points between two expression level values are taken as the split points; Second, we calculate the information gain of the split points respectively and choose the point that has the highest information gain as the boundary. The algorithm of expression boundary identification with information theory is illustrated in Algorithm S1 in Additional file 2.

### GO and KEGG enrichment analysis

The GO enrichment analysis is the major gene-annotation analysis method based on the Gene Ontology resource, describing the gene function at a molecular level. The Kyoto Encyclopedia of Genes and Genomes (KEGG) pathway enrichment analysis has been widely used to model and simulate the molecular interactions and reaction networks in system biology. In this paper, these two methods are applied to figure out the molecular functions of identified potential marker genes and validate whether these genes are related to lung cancer. Here the enrichment analysis methods are both implemented based on the R package called ClusterProfiler developed by Guangchuang Yu’s team [44]. The GO terms and enriched pathways are all filtered with the *p*-value <0.05.

### Conventional DEA method and machine learning evaluation

In this work, two conventional differentially expressed gene analysis methods, t-test and fold change, are compared with the proposed kernel MMD. The t-test is completed based on a python package called ’Scipy’ [45]. The fold change is calculated as below:

8 $$ Fold Change =\left | log_{2} (\frac{E1}{E2})\right |  $$

Where *E1* and *E2* are the average of gene expression level in two different issue types. The *p*-value, fold change value and MMD score are calculated for every single gene in our datasets. Then genes are ranked with the same strategy and top ranking genes are regarded as potential markers. Here a 10-fold cross validation based on the random forest classifier is applied to evaluate the efficiency of these top genes under four frequently used metrics: recall, F1-score, accuracy and Matthews correlation coefficient (MCC).

## Results

In the first part, we present the genes ranking with kernel MMD score and analysis the gene expression difference between different issue types. Then the top ranked genes are reported and compared with those genes identified by conventional t-test and fold change methods. The third part shows the results of GO and KEGG pathway analysis of the top ranked genes. At last, we choose the top ten genes of average ranking as marker gene and identify the expression boundaries of these gene markers with information gain theory.

### Gene differential expression between different tissue types

For the three mentioned datasets, kernel MMD values are calculated on each two of them respectively to discover DEGs. For every single gene, we calculate three MMD values which are from Normal-NAT, Normal-Tumor and NAT-Tumor groups. The MMD scores indicate the difference of expression levels among three types of samples. The top 10 ranked genes in each group are shown in Table 1. As illustrated in the table, the top MMD scores in Normal-Tumor group are over 200, which are much higher than the other two groups. The Normal-NAT group has comparable MMD scores with NAT-Tumor group. It is clear that gene expression level difference in normal-tumor group is much greater than other two groups.

**Table 1:** Top ranking differentially expressing genes between each two tissue types (NAT: Normal Adjacent Tumor)

Ranking	Normal-NAT	MMD scores	Normal-Tumor	MMD scores	NAT-Tumor	MMD scores
1	LOC442459	81.56	LOC442459	300.89	RS1	67.06
2	DOM3Z	70.85	LOC100132831	293.86	C10orf67	58.85
3	LOC100132831	68.89	LOC401127	288.92	ODAM	57.90
4	LOC401127	67.45	PIN1P1	265.11	LOC100128164	57.16
5	CSNK1A1P1	67.02	CSNK1A1P1	264.75	SH3GL3	56.96
6	MKRN9P	66.54	WNT2B	248.53	JPH4	56.68
7	TPI1P2	65.14	LOC100287632	247.69	SGCG	56.56
8	CYP2D7P1	64.72	CSNK1A1L	247.45	GYPE	55.70
9	CSNK1A1L	63.69	LOC100507373	244.54	LOC643650	53.05
10	PIN1P1	62.24	AOC4	240.66	IHH	52.79

In addition, the NAT samples have different expression profiles from not only tumor samples but also the real healthy samples. Since the NAT samples are always considered as healthy samples in the state-of-art researches, we test the top 10 ranked genes selected by NAT-Tumor group, Normal-Tumor group and their average ranking to explore the influence of regarding NAT as real normal samples. To evaluate the effectiveness of selected genes, the expression data of the top genes above is applied to classify tumor samples from other samples via 10-fold cross-validation. The results of the 10-fold cross validation are reported in Table 2.

**Table 2:** Cross Validation Performance of Top Ten genes from different groups (NAT: Normal Adjacent Tumor)

Group	Recall	F1	Accuracy	MCC
Normal-Tumor	0.9857	0.9540	0.9476	0.8659
NAT-Tumor	0.9534	0.9670	0.9640	0.9279
Average	0.9885	0.9914	0.9907	0.9816

As shown in Table 2, the selected genes from each group can classify tumor samples from other samples. However, the performance of the three groups of genes varies greatly. When considering normal samples and NAT samples together, the top average ranked genes have the best scores under all metrics with an accuracy of 0.9907. The highest F1 score of 0.9914 implies that these genes also have a better classification balance. The results show that the real normal samples and NAT samples are not exactly the same. Researchers should take both of them into consideration in cancer study rather than simply replacing real normal samples with NAT samples. The detailed results of differential expressing gene identification conducted by MMD and other two conventional methods are listed in Additional file 1.

### Identify marker genes for lung cancer development

In this work, two conventional DEA methods t-test and fold change are compared with our approach. T-test and fold change methods are both applied to identify DEGs between different tissue types. The *p*-value of t-test and fold change value are calculated to evaluate the gene expression difference. Since the ability to detect tumor samples is more significant in clinical application, the top 10 genes of average rankings from Normal-Tumor group and NAT-tumor group selected by t-test and fold change are compared with the genes selected by our method. Another 10-fold cross validation is conducted and the results are reported in Table 3.

**Table 3:** Cross Validation Performance of top ten genes selected by different DEA methods

Method	Recall	F1	Accuracy	MCC
Fold Change	0.7044	0.7992	0.8048	0.6382
T-test	0.9796	0.9815	0.9794	0.9582
Kernel MMD	0.9885	0.9914	0.9907	0.9816

As shown in Table 3, the proposed kernel MMD method outperforms other two conventional methods under all metrics with the recall of 0.9885, F1 score of 0.9914, accuracy of 0.9907 and MCC of 0.9816. The fold change method has the worst performance and the selected genes by fold change method are not efficient enough to classify tumors from other samples. The t-test has a comparable result with MMD method. Since the t-test and fold change methods have been widely used, the kernel MMD method is promising to improve the differential gene analysis efficiency in practical use.

**Fig. 2:**
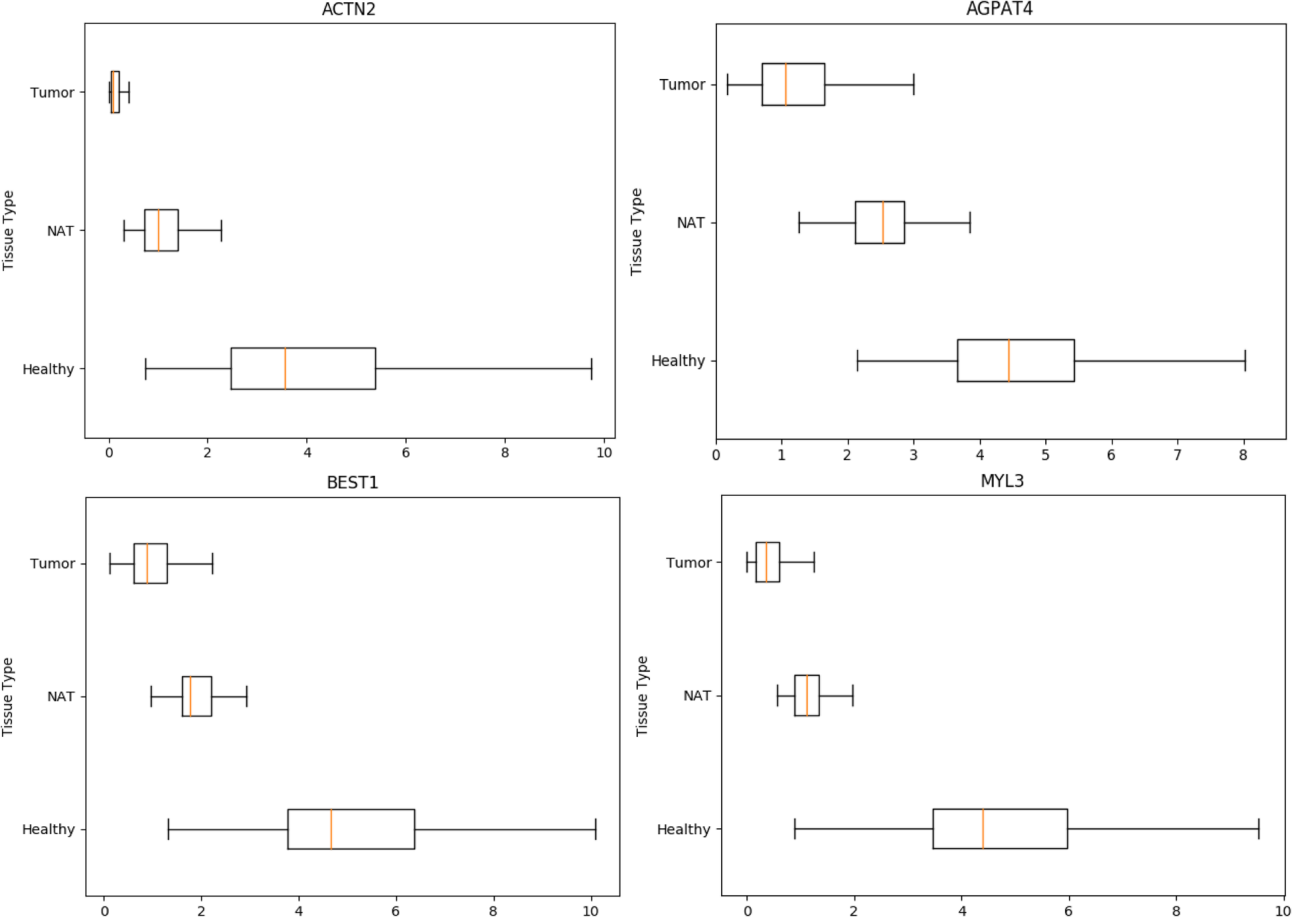
Box-plot of Gene Expression levels in three tissue types.The X-axis is the FPKM expression level; the Y-axis is the tissue type

From Table 1, we can see there are some overlapping genes like LOC442459, LOC100132831, LOC401127, CSNK1A1P1, CSNK1A1L and PIN1P1 in Normal-NAT group and Normal-Tumor group. These genes can distinguish normal samples from not only NAT samples, but also tumor samples. Inspired the previous part, the average ranking of all groups can help to identify more significant genes. Thus, the gene average ranking of the three groups is calculated and top genes of average ranking are chosen to be potential marker genes to diagnose lung cancer. In Fig. 2, expression levels in normal, NAT and tumor samples of the top 4 genes of average ranking are presented. From the figure, the four genes exactly have distinct expression levels in different types of tissues.

### GO and KEGG pathway enrichment

From the average ranking gene list, we choose the top 100 genes to conduct the GO and KEGG pathway enrichment analysis. In the GO enrichment analysis, we select ’Biology Process’ as the enrichment target, and there are 12 GO terms with *p*-value <1.0e-04 and count ≥5. As shown in Table 4, the top two terms, ’GO:0051480’ and ’GO:0007204’, are both related to the regulation factors of cytosolic calcium ion concentration while term No.5 and No.6 are also involved in cellular calcium ion homeostasis. The influence of calcium ion channels on lung cancer has been studied for a long time [46–48], and the cellular calcium ion level change has been explored in lung cancer development [48]. It is suggested that these calcium ion regulation related genes are significant in lung cancer.

**Table 4:** Go Function analysis for the top ranking genes(*p*-value <1.0e-04 and count ≥5)

No.	GOBPID	*p*-Value	Count	Term
1	GO:0051480	7.6032e-07	10	regulation of cytosolic calcium ion concentration
2	GO:0007204	3.0453e-06	9	positive regulation of cytosolic calcium ion concentration
3	GO:0019229	4.4969e-06	5	regulation of vasoconstriction
4	GO:0007200	6.6689e-06	6	phospholipase C-activating G-protein coupled receptor signaling pathway
5	GO:0006874	7.5060e-06	10	cellular calcium ion homeostasis
6	GO:0055074	9.4074e-06	10	calcium ion homeostasis
7	GO:0042310	1.4462e-05	5	vasoconstriction
8	GO:0072503	1.5632e-05	10	cellular divalent inorganic cation homeostasis
9	GO:0072507	2.1785e-05	10	divalent inorganic cation homeostasis
10	GO:0097756	2.3563e-05	5	negative regulation of blood vessel diameter
11	GO:0007189	6.5898e-05	5	adenylate cyclase-activating G-protein coupled receptor signaling pathway
12	GO:0019932	7.4403e-05	8	second-messenger-mediated signaling

**Fig. 3:**
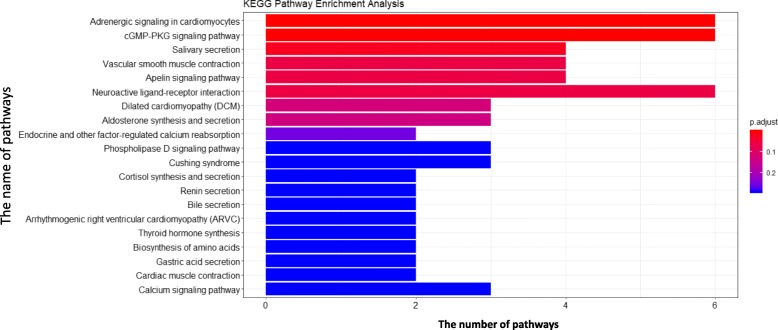
KEGG pathway enrichment analysis for top ranking genes

The results of KEGG pathway enrichment analysis are illustrated in Fig. 3. There are 20 pathways with a *p*-value below 0.05 and count number over 2. The adrenergic signaling pathway and the cGMP-PKG signaling pathway are the most significant pathways. Currently, the role of adrenergic signaling pathways plays in lung cancer development have not been fully studied. However, the *β*-adrenergic signaling have been found to be a possible novel cancer therapy in tumor cells [49]. Besides, some researches have made some explorations about that [50]. The second top significant pathway is the cGMP-PKG signaling pathway which mediates the action of cellular ion concentration and sensitivity, influencing cell proliferation. The regulation relationship between cGMP-PKG signaling pathway and lung cancer has been studied in [51]. The results of GO and KEGG pathway enrichment analysis show that the top gene selected by MMD method is indeed highly related to lung cancer.

### Expression boundary identification

Although the conventional methods can detect the differential expressed gene, they can only manually define the expression boundary when there is a distinct expression level gap. After selecting lung cancer marker genes, we identify the expression boundaries between normal,NAT and tumor with the mentioned information theory method. Here the top 10 genes in MMD average ranking list are chosen as the lung cancer marker genes and the expression boundaries of them are illustrated in Table 5. The identified expression boundaries of all genes are reported in Additional file 1.

**Table 5:** Expression Boundary of Lung Cancer Biomarkers(*e* : FPKM expression level)

Gene Name	Normal	Normal Adjacent Tumor	Tumor
ACTN2	*e* ≥3.5247	0.7146 <*e* <3.5247	*e* ≤0.7146
MYL3	*e* ≥5.3223	4.9211 <*e* <5.3223	*e* ≤4.9211
AGPAT4	*e* ≥4.3722	2.3052 <*e* <4.3722	*e* ≤2.3052
BEST1	*e* ≥3.7487	1.6216 <*e* <3.7487	*e* ≤1.6216
TWIST2	*e* ≥4.2450	1.2030 <*e* <4.2450	*e* ≤1.2030
LINC00472	*e* ≥3.4721	0.8045 <*e* <3.4721	*e* ≤0.8045
MYO7B	*e* ≥4.1723	0.7450 <*e* <4.1723	*e* ≤0.7450
CCNF	*e* ≤16.4506	16.4506 <*e* <20.5656	*e* ≥20.5656
NECAB1	0.9961 <*e* <4.7770	*e* ≥4.7770	*e* ≤0.9961
NOTCH4	*e* ≥4.6829	1.9808 <*e* <4.6829	*e* ≤1.9808

As shown in Table 5, the ten gene markers have a distinct expression range in normal, NAT and tumor samples, which can be an indicator of lung cancer development. Additionally, in practical clinic application, the boundary between tumor and other tissues is the most significant for disease diagnosis.The boundary between normal samples and NAT samples also implied that there would be some gene expression changes in the disease development and the NAT samples may serve to detect cell carcinogenesis, which can help to understand the lung cancer mechanisms.

## Discussion

Since the early-diagnosis of lung cancer has been a long-term critical problem in clinical practice, researchers have explored various types of biomarkers, like genetic mutations, blood proteins. Here, this paper proposed a novel method to identify genes markers for lung cancer. There are two main problems in efficient gene markers identification: first, how to evaluate the gene expression difference; second, how to find the reliable expression boundary between tumor and other samples. The most existing DEA methods were built to solve the first problem, but they can only give out a *p*-value to assess the differential expressing gene without defining the expression boundary. The ln of this research is to address both of the problems in biomarker identifications.

The gene markers are given out based on the existing lung cancer dataset. We think there are two limitations in our work. First, a larger dataset can help to obtain more accurate results; Second, a threshold of MMD value to define the differentially expressed gene can be defined with a large dataset, while here we just take the top ranked genes as potential marker genes.

## Conclusion

In this paper, we not only proposed a more efficient method, kernel MMD, to evaluate the expression changes, but also provide a information theory based algorithm to identify the gene expression boundary. The experiment results show our method can select more significant genes than traditional methods and give out the expression boundary of the marker gene. Through the GO and KEGG pathway enrichment analysis, the function of marker genes in lung cancer is studied, and these marker genes are indeed related to lung cancer development. In the future, we will collect more gene expression data related to lung cancer and calculate more accurate results. In addition, we will explore the application of our method on biomarker discovery for other diseases.

## Supplementary information

**Additional file 1** The results of DEA and boundary identification. In Additional file 1, the results of MMD, t-test and fold change analysis between Normal-NAT, Normal-Tumor and NAT-Tumor are reported. The mmd score, *p*-value and fold change score for every single gene are all presented. Besides, the boundary identification results are also included in this file.

**Additional file 2** The supplementary files. In Additional file 2, the gene differential expression boundary identification algorithm is included.

## Data Availability

The datasets generated and/or analyzed during the current study are available in NCBI Gene Expression Om-nibus (https://www.ncbi.nlm.nih.gov/geo/) under the accession number GSE86354 and GSE62944. The source code of our method is freely available at https://github.com/Zhixun-Zhao/GeneMarker.

## Abbreviations

DEA: Differentially expressed analysis
GO: Gene ontology
KEGG: Kyoto encyclopedia of genes and genomes
MMD: Maximum mean discrepancy
NAT: Normal adjacent to the tumor
