# Supplementary material for: Identification of lung cancer gene markers through kernel maximum mean discrepancy and information entropy
Source: BMC Med Genomics. 2019 Dec 20;12(Suppl 8):183. doi: 10.1186/s12920-019-0630-4 (PMC6923882; doi:10.1186/s12920-019-0630-4)
Supplement: Supplementary file 2 — Additional file 2 The supplementary files. In Additional file 2, the gene differential expression boundary identification algorithm is included. [file 12920_2019_630_MOESM2_ESM.pdf]

Supplementary materials

Algorithm S1. Gene Expression Boundary Identification Algorithm

Algorithm 1: Gene Differential Expression Boundary Identification Algorithm

Input: The gene expression level data E_A and E_B in Group A and Group B; A has m samples and B has n samples. $E_A = (x_1, x_2, \ldots, x_m)$ and $E_B = (y_1, y_2, \ldots, y_n)$.

1: Combine E_A and E_B into a new vector and sort all values. The new established vector $E = (e_1, e_2, \ldots, e_{n+m})$.

2: **for** $i = 1$ to $m+n$ **do**

3: $S[\mathrm{i}] = (e_i + e_{i+1}) / 2$

4: **end for**

5: Obtain the split point sets $S = (s_1, s_2, \ldots, s_{m+n-1})$

6: Calculate the original Entropy $\mathbf{En} = \sum_{c \in C} \boldsymbol{P(c) log_2 P(c)}$
where C refers to Group A and B, and P(c) is the sample ratio.

7: **for** s_i in S **do**

8: **for** e_i in E **do**

9: if $e_i < s_i$:

10: if $e_i \in$ A:

11: True A += 1

12: else:

13: False A += 1

14: else:

15: if $e_i \in$ A:

16: False B += 1

17: else:

18: True B += 1

19: **end for**

20: $\mathbf{En}_i = \sum_{c \in C} \boldsymbol{P(c) log_2 P(c)}$
where C refer to True A, false A, True B and False B and P(c) is the sample ratio.

21: **end for**

22: The s_i with the largest En_i is the best split point

Output: *The best split point (Boundary) of Gene Expression*
